# Analysis of translesion polymerases in colorectal cancer cells following cetuximab treatment: A network perspective

**DOI:** 10.1002/cam4.6945

**Published:** 2024-01-25

**Authors:** Anubrata Das, Georgios V. Gkoutos, Animesh Acharjee

**Affiliations:** ^1^ Institute of Cancer and Genomic Sciences, College of Medical and Dental Sciences University of Birmingham Birmingham UK; ^2^ Institute of Translational Medicine University Hospitals Birmingham NHS Foundation Trust Birmingham UK; ^3^ MRC Health Data Research UK (HDR UK) London UK; ^4^ Centre for Health Data Research University of Birmingham Birmingham UK; ^5^ NIHR Experimental Cancer Medicine Centre Birmingham UK

**Keywords:** cancer, cetuximab, mutagenesis, network, TLS polymerase

## Abstract

**Introduction:**

Adaptive mutagenesis observed in colorectal cancer (CRC) cells upon exposure to EGFR inhibitors contributes to the development of resistance and recurrence. Multiple investigations have indicated a parallel between cancer cells and bacteria in terms of exhibiting adaptive mutagenesis. This phenomenon entails a transient and coordinated escalation of error‐prone translesion synthesis polymerases (TLS polymerases), resulting in mutagenesis of a magnitude sufficient to drive the selection of resistant phenotypes.

**Methods:**

In this study, we conducted a comprehensive pan‐transcriptome analysis of the regulatory framework within CRC cells, with the objective of identifying potential transcriptome modules encompassing certain translesion polymerases and the associated transcription factors (TFs) that govern them. Our sampling strategy involved the collection of transcriptomic data from tumors treated with cetuximab, an EGFR inhibitor, untreated CRC tumors, and colorectal‐derived cell lines, resulting in a diverse dataset. Subsequently, we identified co‐regulated modules using weighted correlation network analysis with a minKMEtostay threshold set at 0.5 to minimize false‐positive module identifications and mapped the modules to STRING annotations. Furthermore, we explored the putative TFs influencing these modules using KBoost, a kernel PCA regression model.

**Results:**

Our analysis did not reveal a distinct transcriptional profile specific to cetuximab treatment. Moreover, we elucidated co‐expression modules housing genes, for example, POLK, POLI, POLQ, REV1, POLN, and POLM. Specifically, POLK, POLI, and POLQ were assigned to the “blue” module, which also encompassed critical DNA damage response enzymes, for example. BRCA1, BRCA2, MSH6, and MSH2. To delineate the transcriptional control of this module, we investigated associated TFs, highlighting the roles of prominent cancer‐associated TFs, such as CENPA, HNF1A, and E2F7.

**Conclusion:**

We found that translesion polymerases are co‐regulated with DNA mismatch repair and cell cycle‐associated factors. We did not, however, identified any networks specific to cetuximab treatment indicating that the response to EGFR inhibitors relates to a general stress response mechanism.

## Graphical abstract

Overview of the workflow.

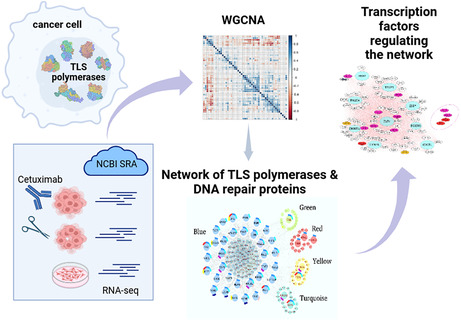

## 1 INTRODUCTION

In 2018, the global incidence of colorectal cancer (CRC) stood at 1.8 million cases, resulting in 880,792 fatalities.^1^ CRC continues to pose a significant health burden, particularly in developed regions.^2^ Its risk factors can be categorized into modifiable and nonmodifiable elements. Nonmodifiable factors encompass age, race, and genetically predisposing mutations, while modifiable factors encompass aspects such as diet, smoking, and alcohol consumption tendencies, and levels of physical activity.^3^ Over the years, advancements in screening and diagnostic methodologies, coupled with the integration of neoadjuvant chemotherapy and radiotherapy protocols prior to surgical interventions, have substantially ameliorated the severity of this ailment.^4^

CRC manifests as a consequence of specific deficiencies within the DNA repair machinery, critically involving microsatellite instability (MSI) cancers.^5^ These MSI cancers arise due to mutations within the mismatch repair system (MMR), primarily implicating a number of proteins, such as MLH1, PMS2, MSH2, and MSH6.^6^ Consequently, these tumors exhibit elevated tumor mutation burden, facilitating heightened immune system responsiveness.^7^ Furthermore, key DNA repair enzymes, namely PARP‐1 and BRCA‐1, exert significant influence on CRC by governing essential processes, such as DNA repair, DNA replication, chromatin dynamics, and mitotic processes. Recent research has shown that even ncRNA plays an important role in this aspect.^8^ The interplay between PARP‐1 and BRCA‐1, rescuing defects each other, has been leveraged to develop PARP inhibitors (PARPi) targeting homologous recombination deficient cancers, including CRC. By inhibiting PARP‐1 activity, these inhibitors incapacitate DNA repair mechanisms, leading to the eradication of such cancers.^9^ Translesion polymerases (TLS polymerases) can synthesize past damaged DNA and prevent the stalling of replication forks and form an important component of the DNA repair mechanism.^10^ There are nine known translesion polymerases but the regulatory networks which govern their activity is not very well understood.^11^ There is mounting evidence that dysregulated polymerases contribute to carcinogenesis^12^ and hence it is worthwhile to investigate the putative networks governing their activity.

As highlighted earlier, mutations in DNA repair genes significantly impact CRC. Notably, EGFR inhibitor treatment drives mutations in KRAS and NRAS, as well as in the extracellular domains of EGFR, imparting resistance to targeted therapy.^13^ Particularly pertinent is the activation of an “adaptive response” following targeted therapies, wherein DNA repair pathways are suppressed while mutagenic pathways are concurrently stimulated. Pioneering work by Bardelli et al. revealed that cetuximab‐treated CRC cells exhibit simultaneous downregulation of the MMR system and upregulation of error‐prone translesion polymerases, underscoring the persistence and complexity of this phenomenon.^14^ Furthermore, evidence suggests the activation of comprehensive pathways, encompassing the overexpression of RAD6/RAD18 ubiquitination enzymes and increased PCNA ubiquitination, further implicating the intricacies of this adaptive response.^15^

In this study, we systematically analyzed publicly available transcriptomic datasets encompassing CRC cells exposed to cetuximab, untreated cells, and cell lines not exposed to the agent. Our objective was to delineate the regulatory and interaction network involving TLS polymerases and DNA repair enzymes within this context. Achieving this objective can potentially advance our understanding of adaptive responses and resistance mechanisms against targeted therapies, potentially paving the way for proactive strategies to mitigate these challenges.

## 2 MATERIALS AND METHODS

### 2.1 Public data collection

**FIGURE 1:**
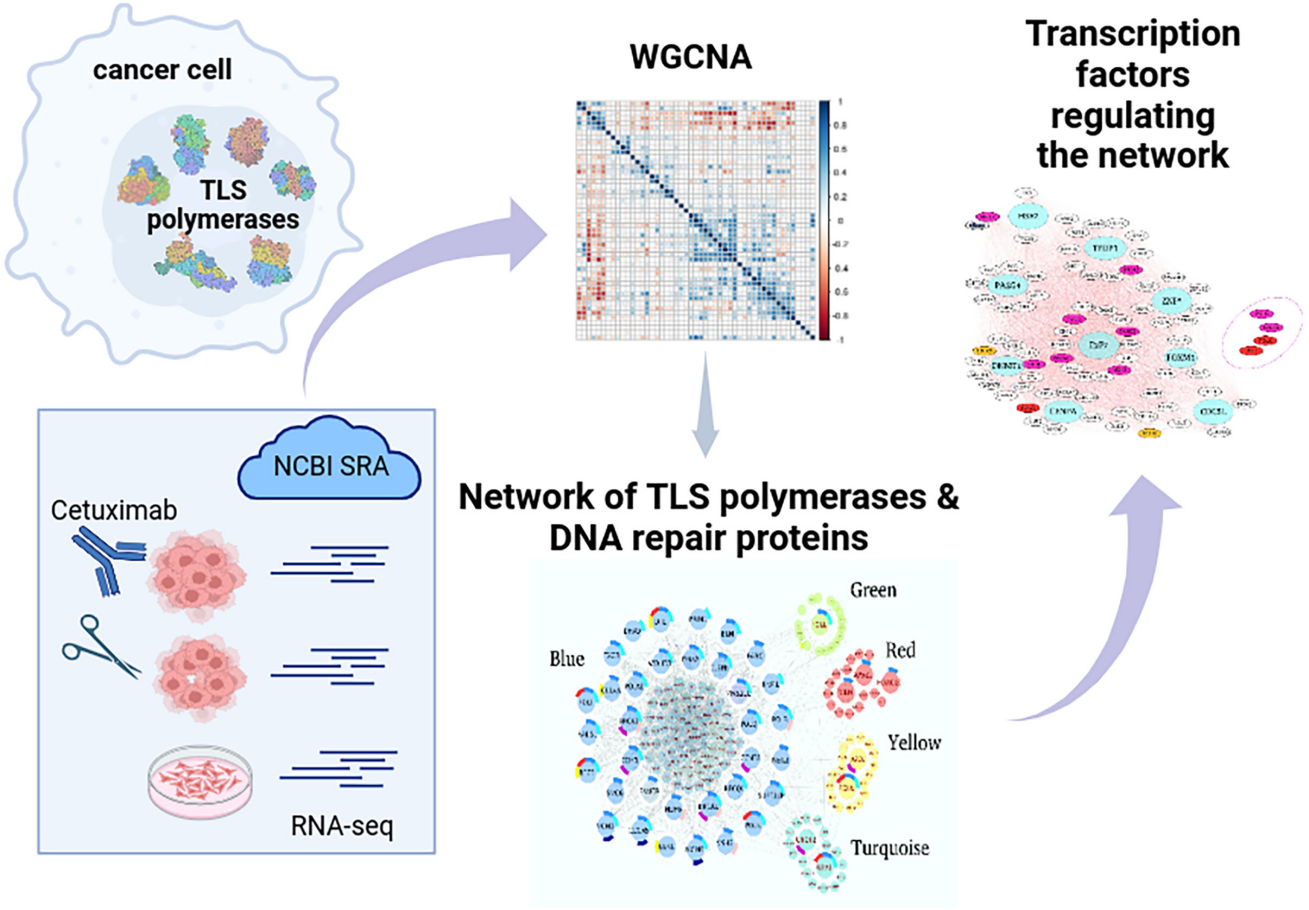
Graphical abstract of the workflow.

Expression profiles of surgically excised colorectal tumor tissue (GSE156451),^16^ cell lines of colorectal origin (HT29, LS513, LS174T, and HCT116) (GSE185055)^17^ and cetuximab‐treated CRC tissue (GSE196576),^18^, ^19^ were obtained from the Gene Expression Omnibus (GEO) (https://www.ncbi.nlm.nih.gov/geo/) on March 21, 2023. The details can be found in the Table 1. The resulting Illumina sequencing dataset includes RNA‐Seq profiles of 150 samples from 50 tumor tissue samples, 50 cell line samples, and 50 cetuximab‐treated samples. Count‐based reads in the samples were converted to fragments per kilobase million (FPKM) values for within‐sample normalization. The gene lengths were obtained from the ENSEMBL BioMart and the FPKM values were calculated using the DESeq2 v1.20 package in R (version 4.2.3). Subsequently, the FPKM values of all the samples were quantile normalized by quantile function in R to ensure identical sample distributions. The ComBat‐Seq package (Bioconductor sva v3.36.0) was used to normalize batch effects, and the PCA plots were produced to verify the absence of batch effects.^20^ Following the normalization of read counts of the RNA‐Seq data, the values of FPKM for each gene were used for the subsequent analysis. A graphical abstract of the workflow is presented in the Figure 1.

**TABLE 1:** List of the Gene Expression Omnibus (GEO) RNA sequence datasets are described. The datasets in GEO short read archives (SRA) which store FASTQ data from which the data were collected.

Sample	Assay type	Library layout	SRA study	Source name
GSE156451	RNA‐Seq	Paired	SRP278056	Tumor from colorectal cancer (CRC) patient
GSE196576	RNA‐Seq	Paired	SRP359396	Cetuximab treated CRC patient
GSE185055	RNA‐Seq	Single	SRP339453	CRC cells grown in 2D/3D conditions

### 2.2 Colorectal Cancer co‐expression module analysis

Weighted gene co‐expression network analysis (WGCNA) was used to investigate co‐expression modules related to CRC.^21^ The adjacency in WGCNA is based on the soft‐threshold power (*β*) which is determined by two parameters, namely scale independence and mean connectivity. *β* value changes was plotted w.r.t scale independence and mean connectivity. The *β* of 7 showed *R* ^2^ value of 0.9 for scale independence and a mean connectivity of 30, beyond which these values remained constant (Figure S3). Hence the co‐expression matrix was then calculated with a *β* of 7.The minimal module size was set to 30 and additionally the merge cut height was chosen as 0.5 to select for modules with at least 30 members. The network was signed to select positive correlations and the spurious membership was reduced with minKMEtostay set at 0.5. WCGNA labels each individual module of the discovered network with a unique color and the largest modules are labeled turquoise, blue, brown, yellow, green, red, black, etc.

### 2.3 Module–trait analysis based on characteristics of cell origin

The module–trait relationships within the tissue expression dataset were investigated for correlation with cetuximab treatment. The sample characteristics (tumor, cell line, and cetuximab) were binarized to generate a trait matrix by creating a 150 × 3 numerical matrix and labelling the sample corresponding to its specific expression profile as one with the other two profiles labeled as 0.We used Kendall rank correlation to find the correlation between the eigengenes obtained from WGCNA with the trait data for the samples provided at SRA. We visualized the above relationship with a heat map. Additionally, binomial logistic regression models were created for each eigengene within the cetuximab‐treated sample set so as to obtain statistically relevant *β* coefficients. Finally, the distribution of these eigengenes was visualized using the lattice library (version 0.20.45) in R (version 4.2.3).

### 2.4 Mapping of co‐expressing modules to STRING network data

We assembled a set comprising TLS polymerases and repair proteins, previously identified to be differentially regulated by Bardelli et al.^14^ designated by HGNC codes: POLH, POLI, POLK, POLL, POLM, POLQ, POLZ, REV1, POLN, POLA1, POLB, POLG1, POLD, POLDE, MLH1, MSH2, MSH6, BRCA2, RAD51, and EXO1. We systematically scanned the modules generated by WGCNA for the presence of these genes. Notably, certain TLS polymerases, such as POLH, were absent from all modules (except the grey module, which encompasses unclustered genes). Using an adjacency matrix constructed in WGCNA, we extracted the 10 nearest neighbors of each of the genes identified as present within the modules and generated a set of 209 proteins. Subsequently, we employed Cytoscape, an open‐source software specializing in analyzing and visualizing interactions among data points particularly suited for interpreting high‐throughput data outcomes such as RNA‐Seq,^22^ and used the resulting protein set to interrogate the STRING database. STRING is a comprehensive protein–protein network database, utilizing both experimental data and predictions to map interactions.^23^ The identified proteins were then categorized module‐wise and annotated using the STRING server plugin.

### 2.5 Transcription Factors acting on TLS polymerases

We used KBoost^24^ to discover the transcription factors (TFs) interacting with the TLS polymerases using our gene expression dataset. KBoost employs an algorithm that uses kernel PCA regression, boosting and Bayesian model averaging for fast and accurate reconstruction of gene regulatory networks. This algorithm performed favorably against the benchmark IRMA and DREAM4 datasets.^25^ We queried our dataset with the *Kboost_human_symbol* function using the standard set of TFs available in the algorithm. The probability matrix returned by the function was used to plot a scree plot of the distribution of the number of predicted targets by the number of TFs. As we had earlier chosen the minimum module size as 30, from the output of KBoost, we chose those TFs which were predicted to act on at least 30 or more targets (^26^).For choosing reliable TF‐target relationships, we plotted the distribution of probabilities provided by KBoost for each TF of this subset. We chose only those targets with a probability score >0.3, as we found that this score has a *Z* > 10, which is well above the threshold of three standard deviations. We obtained the nearest neighbors of the chosen TLS polymerases from the sorted adjacency matrix resulting from the expression dataset. Finally, the TFs probability matrix was interrogated for the presence of the chosen TLS polymerases and their neighbors as putative targets.

## 3 RESULTS

### 3.1 Transcription modules reveal a poor correlation with cetuximab treatment‐specific cell origin

To study the regulatory network of the CRC cell adaptive response in response to targeted therapy, we obtained the following publicly available GEO transcriptome datasets, GSE156451, GSE185055, and GSE196576. We then compared the transcriptome of cetuximab‐treated cancer tissue along with CRC cells of similar genetic background albeit exposed to different environments so as to analyze transcriptional response differences to cetuximab. Based on similar studies as well as for reasons of computational tractability, we restricted the sample subsets to 50 randomly resampled cases. We selected 50 representative samples from the 94 CRC samples available from a study by Tao et al.^16^ examining the epigenetic changes associated with CRC in surgically excised tumor tissue without chemotherapy or radiotherapy treatment. Pramil et al.^17^ studied the response of cultured CRC cells to extracellular ATP in four CRC cell lines. HT29 and LS513 were MSS (microsatellite stable) cell lines whereas LS174T and HCT116 were MSI cell lines. We selected 10 cases of HCT116, 18 cases of HT29, 13 cases of LS174T, and 9 cases of HCT116 as representative samples from the 194 samples in the dataset. Vincent et al.^18^ studied the immunogenic response of cetuximab‐ and bevacizumab‐treated CRC tissue. From the 216 cetuximab samples, we selected 50 representative samples. Thus, we built a cohort of 150 samples, consisting of 50 cases each of (a) cetuximab‐treated CRC cells, (b) untreated human tissue, and (c) untreated cell lines.

**FIGURE 2:**
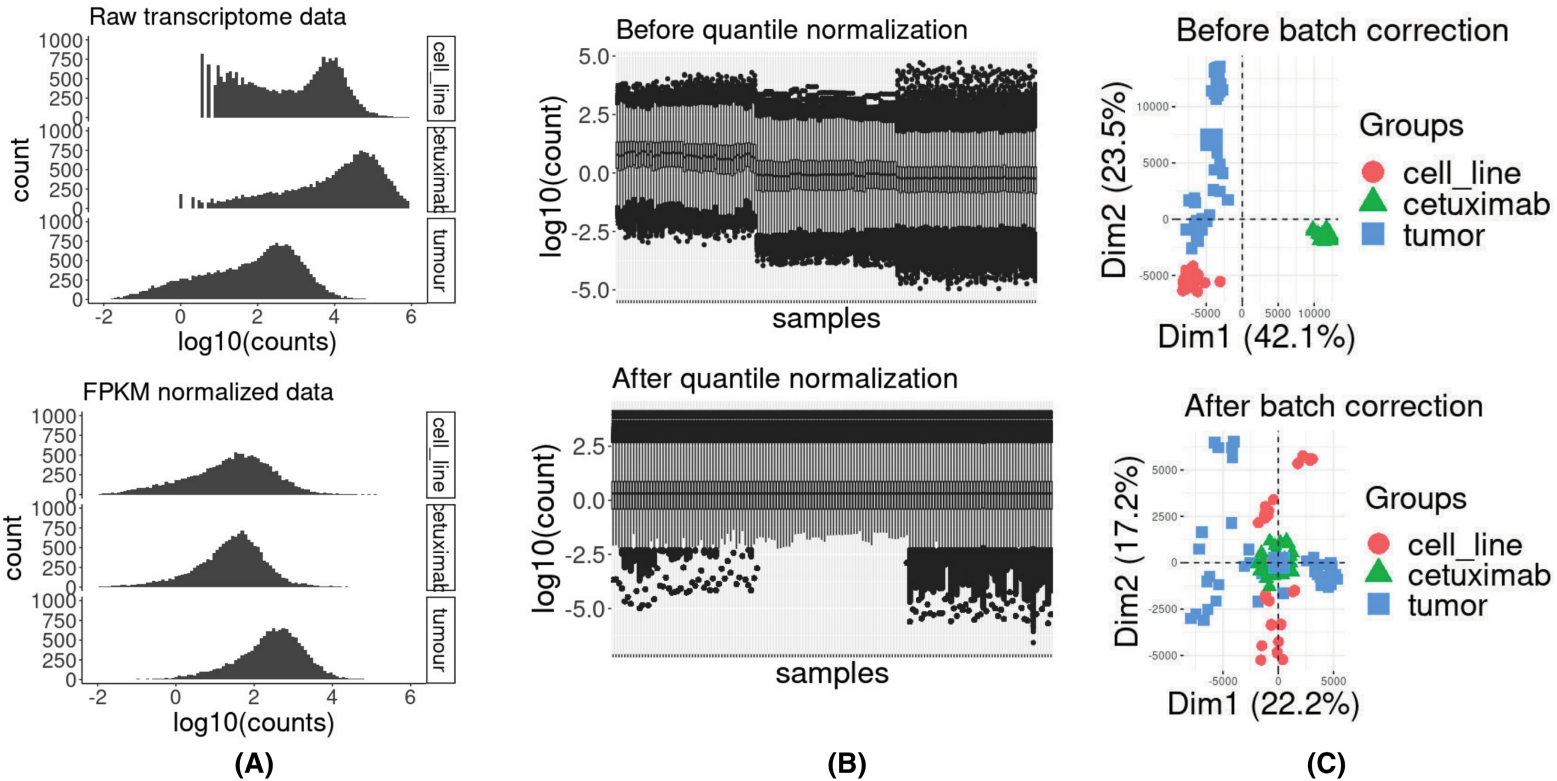
Data normalization flowchart. (A) Distributions of raw transcriptomics and fragments per kilobase million (FPKM) normalized data, (B) distributions of raw transcriptomics and after quantile normalization of the samples, and (C) PCA score plot analysis before and after batch correction of the data.

**FIGURE 3:**
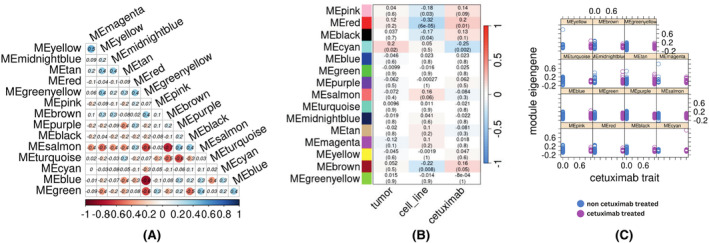
Discovery of co‐expressing modules in the transcriptome dataset. (A) Heatmap of the correlation between the eigengenes of the modules. (B) Module–trait heatmap of the eigengenes and the cell source origin, (C) comparison of the distribution of values of module eigengenes between cetuximab‐treated and ‐untreated samples.

Following normalization within samples (Figure 2A), between samples (Figure 2B), and across batches (Figure 2C), a unified dataset encompassing 150 samples and comprising 15,269 common genes was generated. Subsequently, WGCNA was applied with the previously outlined parameters, leading to the computation of eigengenes. 5439 genes did not align with any modules based on our established parameters. Notably, Pol eta (POLH) was not identified as clustering within any of the modules. Overall, the analysis yielded 15 modules, with the Blue module being the largest, consisting of 2011 members, and the Midnightblue module being the smallest, comprising 73 members. In order to gain insights into the interplay between these modules, we conducted an investigation into the correlation of eigengenes among them (Figure 3A). While the Magenta and Yellow modules revealed a weak positive correlation of (0.48 *p*‐value 3.5e–10), three other modules, namely Greenyellow and Blue (−0.77 *p*‐value 0.0), Brown and Salmon (−0.7 *p*‐value 0.0), and Turquoise and Purple (−0.63 *p*‐value 0.0), exhibited a strong negative correlation between each pair, respectively. We then mapped the genes across these modules onto pathways following an overrepresentation analysis using the g:Profiler suite.^27^ The resulting negative correlated GO enrichment terms associated with each module were then statistically compared to the overall association of all human genes (Figure S1). Nine genes in the Greenyellow module, namely, FBXW4, KLHL22, ANAPC2, DCAF5, CUL9, LZTR1, FBXL15, KCTD13, and SPSB3 were associated with the Cullin–Ring ubiquitin ligase complex (p.adj 9.4 e‐4). Cullin–Ring ubiquitin ligases play a central role in cell cycle and DNA repair.^28^ The Greenyellow module is negatively associated with the Blue module with high statistical significance (p.adj 3.23 e‐42) and the Blue module has several key members of chromosome segregation and cell cycle such as BRCA1, TOP2A, RAD51C, SMC3, TOP1, CDC16, and NEK2 and also has several key DNA repair proteins such as BRCA2, PARP1, FANCM, RAD51, BLM, and PALB2. The Turquoise module includes several genes involved in lipid metabolism, while the Purple module, which is negatively correlated with it, contains genes involved in ribosome biogenesis, an energy driven process negatively regulating lipid metabolism.^29^ Genes involved in fatty acid and lipid metabolism play a key role in carcinogenesis. An earlier study had found that *ACADS* and *DHRS11* genes involved in fatty acid metabolism were differentially expressed in CRC cells and could distinguish between healthy and CRC cell.^30^ We could not explore the relationship of Brown and Salmon module due to the Salmon module being poorly annotated.

In an effort to unveil any correlation between targeted therapy and the discovered modules, we examined the correlation between the eigengenes and the three binarized traits, namely, tumor, cell line, and cetuximab. Using Kendall's rank correlation, we found that some modules had a weak correlation with the targeted therapy phenotype (Figure 3B). The Cyan module was characterized by a weak, probably due to the presence of an outlier in the eigengene value distribution, yet statistically significant correlation with the targeted therapy trait (Figure 3C).

### 3.2 TLS polymerases belong to co‐expression modules having members involved in DNA damage response

**FIGURE 4:**
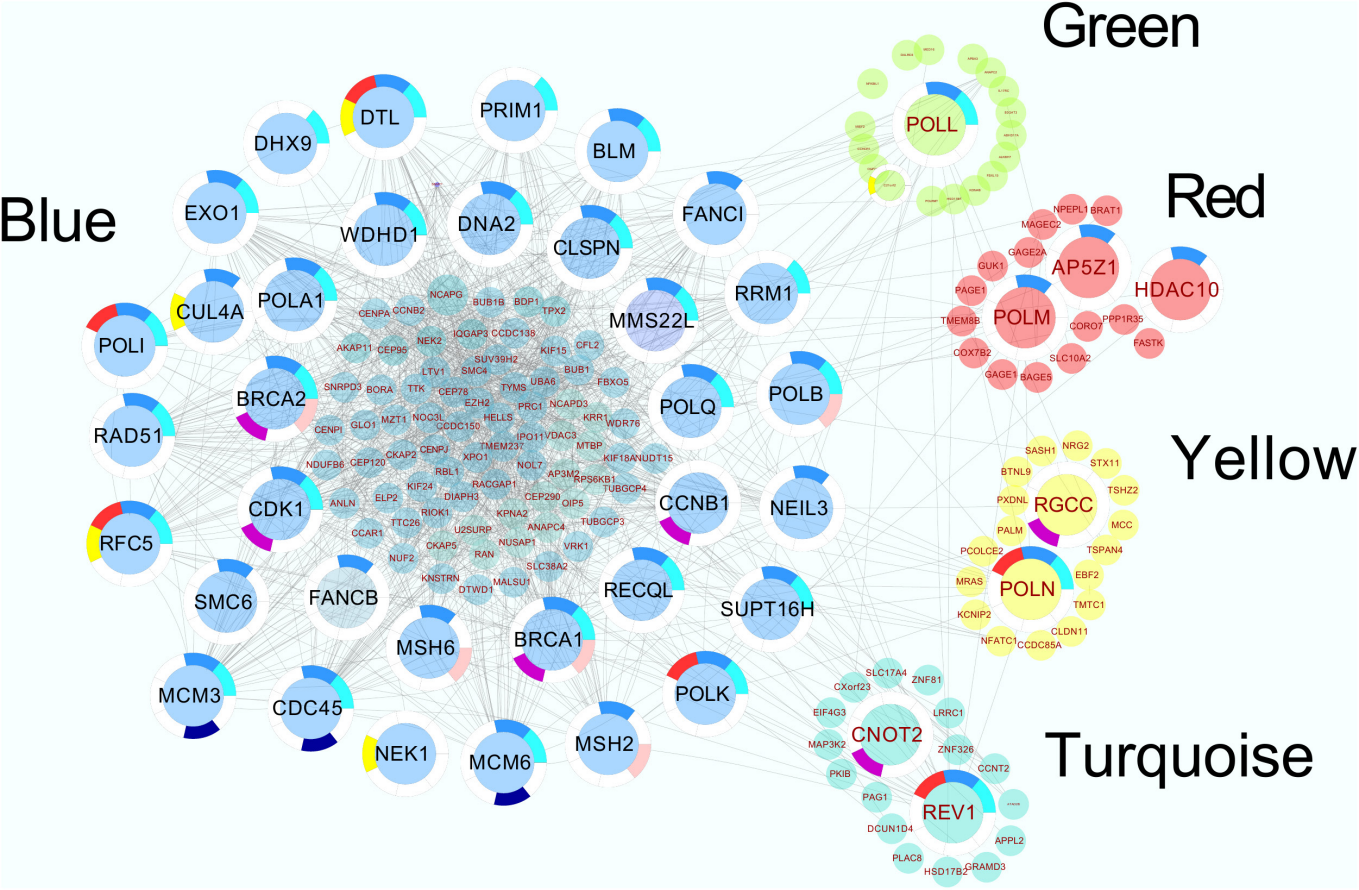
Co‐expression patterns are charted and visualized using Cytoscape, revealing a complex landscape of co‐expression. Individual proteins were annotated using STRING, and the colors used in the visualization corresponds to the modules in which the respective genes were identified.

Figure 4 depicts the distribution of TLS polymerases and their associated genes, with the majority (10 members) located within the Blue module. Additionally, there is one member within the Greenyellow, Red, Yellow, and Turquoise modules. We assessed the strength of the module membership of these polymerases within the largest module, that is, the Blue module, by correlating the expression profiles with the module eigengene. The resulting correlation scores for specific genes were as follows: POLB (0.3), POLA1 (0.64), POLI (0.25), POLQ (0.52), POLK (0.54), EXOI (0.60), BRCA2 (0.45), RAD51 (0.44), MSH2 (0.76), and MSH6 (0.70). In order to enhance the robustness of our network analysis, we cross‐verified our findings by retrieving network data and annotation information from the STRING database for these genes. The STRING server data corroborated the extensive interconnections observed in our analysis using expression data from our dataset. We used Cytoscape to visualize the complex and rich interaction between the TLS polymerases and other genes in the network (Figure 4). The gene network revealed a scale free topology with hubs having multiple interactions with key DNA damage response (DDR) genes, for example, BRCA1, BRCA2, and MSH6 acting as hubs. The TLS polymerases are well integrated within this DDR damage dominant hub and are part of networks entailing other repair proteins. Employing the diffusion function within Cytoscape, we unveiled the network connections of POLI, which interconnects with POLK, POLB, DNMT1, BDP1, BRCA2, MSH6, and others. POLI is experimentally known to interact with PCNA, a member of the Blue module.^31^, ^32^ POLK exhibits a network with partial overlap and additionally interfaces with RECQL, NEIL3, EXO1, and BLM. Similarly, POLQ, interacting with FANCD, manifests its own distinct network. Furthermore, TLS polymerases, located in modules other than the Blue module, also exhibit interactions with genes within the Blue module. Notably, in the Yellow module, POLN, known to interact with BRCA1,^33^ interacts with genes in the Blue module, along with REV1 in the Turquoise module. The profound interaction observed between TLS polymerases and the homologous recombination (HR) pathway is remarkable, considering that these pathways are typically distinct in terms of mechanistic function.

### 3.3 TLS polymerases and their transcriptionally adjacent genes are controlled by TFs prominent in carcinogenesis

**FIGURE 5:**
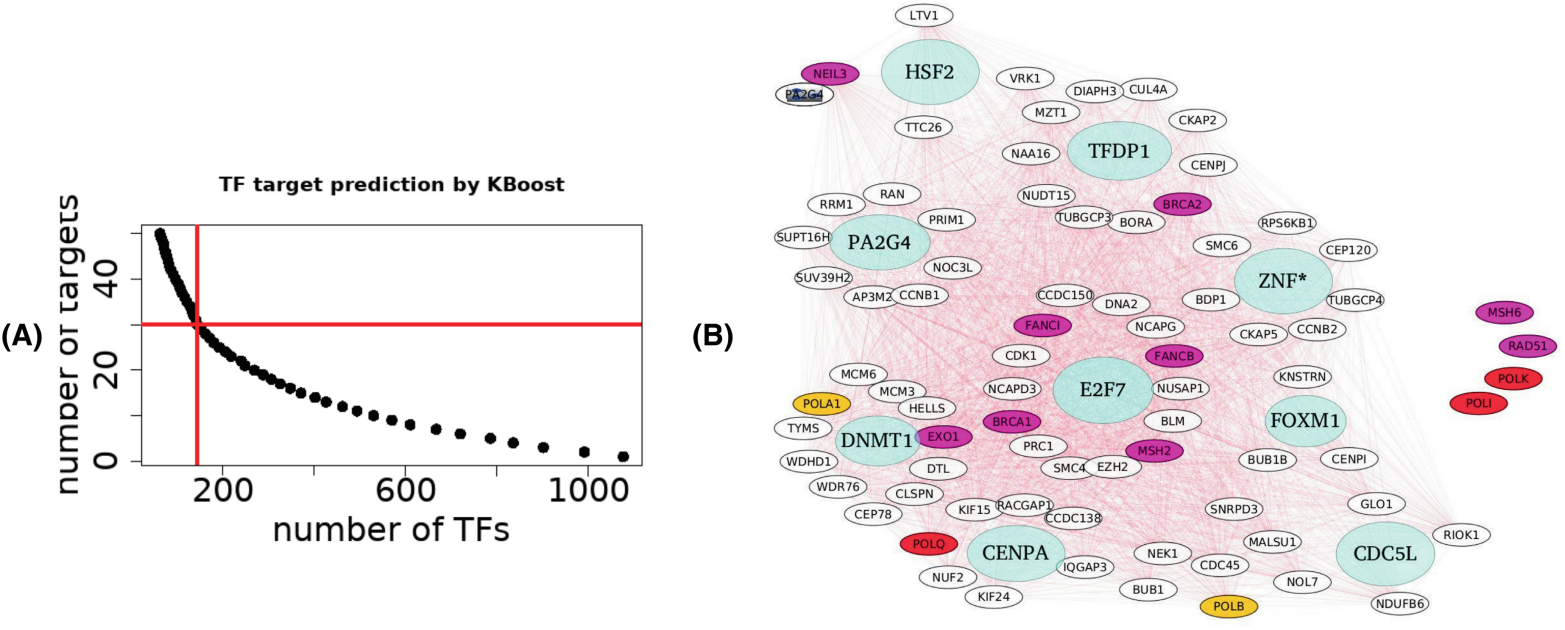
Transcription factors (TFs') target protein interaction. (A) Screeplot of number of TFs versus number of predicted targets. (B) An integrated map of the TFs and the targets in the Blue module.

One of our objectives was to identify the pivotal TFs orchestrating the response of repair polymerases. To achieve this, we employed a robust algorithm, KBoost, utilizing Kernel PCA regression, boosting, and Bayesian analysis.^24^ KBoost enabled us to systematically scan for TFs that influence the network of repair polymerases. Utilizing a scree plot, we determined a subset of TFs that affect 30 or more targets (Figure 5A) and obtained 50 TFs. We proceeded to plot the prediction probability threshold for each TF targets (Figure S2) and selected all targets with a probability exceeding 0.26 (equivalent to 4 standard deviations). Subsequently, we scrutinized this subset for an overlap with genes within the Blue module. Our investigation revealed that numerous pivotal cancer‐responsive TFs are predicted to interact with TLS polymerases (Figure 5B). Notably, CENP‐A, a histone H3 variant crucial for centromere stabilization and frequently overexpressed in cancer,^34^ featured prominently in these predictions. CENPA is predicted to interact with POLQ (*p* = 0.4), which is involved in Interstrand crosslink repair. ZNF644 is a TF involved in site‐specific histone methylation and DNA stabilization^35^ and interacts with SMC6 (*p* = 0.4), BDP1 (*p* = 0.4), and CEP120 (*p* = 0.4). E2F7, another well characterized TF involved in progression of cancer^36^ interacts with SMC4 (*p* = 0.55). All these proteins are close neighbors of POLK. Likewise, POLI interacts with NEK1, a kinase which separates repair from replication and allows for proper repair. NEK1, in turn is predicted to be acted upon by HNF1A (*p* = 0.4), a TF involved synthesis of liver specific transcripts and whose absence predisposes to adenomas.^37^ Thus, the TLS polymerases and associated genes are acted upon by multiple TFs and in a complex circuitry in cancer cells.

## 4 DISCUSSION

Stress‐induced mutagenesis is a well‐studied phenomenon in bacteria. DNA damage and starvation, induces the SOS response, where double strand break repair is carried out by mutagenic polymerases DNA Pol IV, V, and II.^38^ Cancer cells are under metabolic stress due to demands of constant growth and dysregulation.^39^ An example of stress mediated mutagenesis would be the erroneous activation of APOBEC3 enzyme, which mutagenizes by cytosine deamination and causes 75% of kataegis.^40^ Another example of stress‐induced mutagenesis is presented by double strand break inducing cancer therapies, such as etoposide and doxorubicin, which are likely to provoke stress response–dependent error‐prone repair mechanisms.^41^ Cetuximab treatment is an effective therapy against *KRAS/NRAS* CRC, however, resistance inevitably emerges. Bardelli et al. had shown how cetuximab‐treated CRC cell lines have transiently higher levels of mutagenic polymerases and lower levels of MMR proteins^14^ while another study on COSMIC signatures associated with cetuximab‐treated CRC showed no significant increase in SBS3 or SBS6 or SBS15 signatures, which are associated with loss of MMR activity.^42^ Moreover, an earlier transcriptome study on PCNA and TLS polymerases^43^ also identified a co‐regulation of DNA repair genes and TLS polymerases. These findings drove our exploration of whether a cetuximab‐specific transcriptional response exists as well as whether mutagenic polymerases and MMR proteins are indeed co‐regulated.

Our analysis of the transcriptional profile of a sizable number of CRC samples exposed to cetuximab did not reveal any Cetuximab treatment specific transcription module, which might be due to the transient nature of treatment‐induced mutagenesis or to the CRC heterogeneity.^44^ CRC is known to evolve via multiple clones and scRNA‐Seq has revealed that different clones exhibit different transcriptional profiles, for example, CRC bulk RNA‐Seq data have been used to distinguish CRC into four sub‐categories.^45^ When a cell line was treated with cetuximab,^14^ western blots showed increased levels of TLS polymerases. But in our bulk RNA‐Seq analysis of human tissue, we did not find any cetuximab‐specific response, which could be due to the lack of resolution in bulk analysis. A previous study employing ScRNA‐Seq analysis distinguished drug resistant clones from drug‐sensitive clones^46^ and our results point to the need for further scRNA‐Seq analysis to uncover the cell types displaying adaptive mutagenesis. Using fairly rigorous criteria, we have discovered that mutagenic polymerases are part of regulatory modules that form key DNA repair pathways regulatory modules impacted by common TFs. Presumably, the repair pathway choices, as well as the immediate response to external stimuli, occur at the posttranscriptional level. Another important factor modulating transcriptional response lies with the external feedback received from microbiome and metabolome factors associated with CRC.^47^ The limitations of our study include the lack of resolution from bulk RNA‐Seq data due to tumor heterogeneity, as well as the employment of datasets derived from different experiments. Therefore we consider future scRNA‐Seq experiments using common cellular origin and experimental conditions necessary. Despite these limitations, our meta‐transcriptome study reveals that several TLS polymerases are co‐located within the same intricate regulatory network similarly to other DNA repair proteins and should therefore adhere to similar expression patterns to other proteins regulated by the TFs discovered. Given this is an intricate network, involving epigenetic states and clonal evolution, any further study to dissect the regulatory framework or to discover candidate drugs would need to account for these findings.

## AUTHOR CONTRIBUTIONS

**Anubrata Das:** Conceptualization (lead); data curation (lead); formal analysis (lead); investigation (equal); methodology (equal); software (equal); visualization (lead); writing – original draft (equal); writing – review and editing (equal). **Georgios V. Gkoutos:** Funding acquisition (equal); methodology (supporting); supervision (supporting); writing – original draft (supporting); writing – review and editing (equal). **Animesh Acharjee:** Conceptualization (equal); funding acquisition (equal); investigation (lead); methodology (lead); project administration (lead); resources (equal); supervision (equal); writing – original draft (equal); writing – review and editing (equal).

## FUNDING INFORMATION

The authors acknowledge support from the HYPERMARKER (Grant agreement ID 101095480), MAESTRIA (Grant agreement ID 965286), the MRC Heath Data Research UK (HDRUK/CFC/01), Nanocommons H2020‐EU (731032), NIHR Birmingham Surgical Reconstruction Microbiology Research Centre, and Wellcome Leap Inc (Grant agreement ID 2515692). The views expressed in this publication are those of the authors and not necessarily those of the NHS, the National Institute for Health Research, the Medical Research Council, or the Department of Health.

## CONFLICT OF INTEREST STATEMENT

The authors declare no conflict of interest.

## ETHICS STATEMENT

Not applicable.

## CONSENT

Not applicable.

## Supporting information


Figure S1.

Figure S2.

Figure S3.

## Data Availability

All the data used in this study are publicly available for free.
