# Supplementary figures and images for: Analysis of translesion polymerases in colorectal cancer cells following cetuximab treatment: A network perspective
Source: Cancer Med. 2024 Jan 25;13(1):e6945. doi: 10.1002/cam4.6945 (PMC10809876; doi:10.1002/cam4.6945)

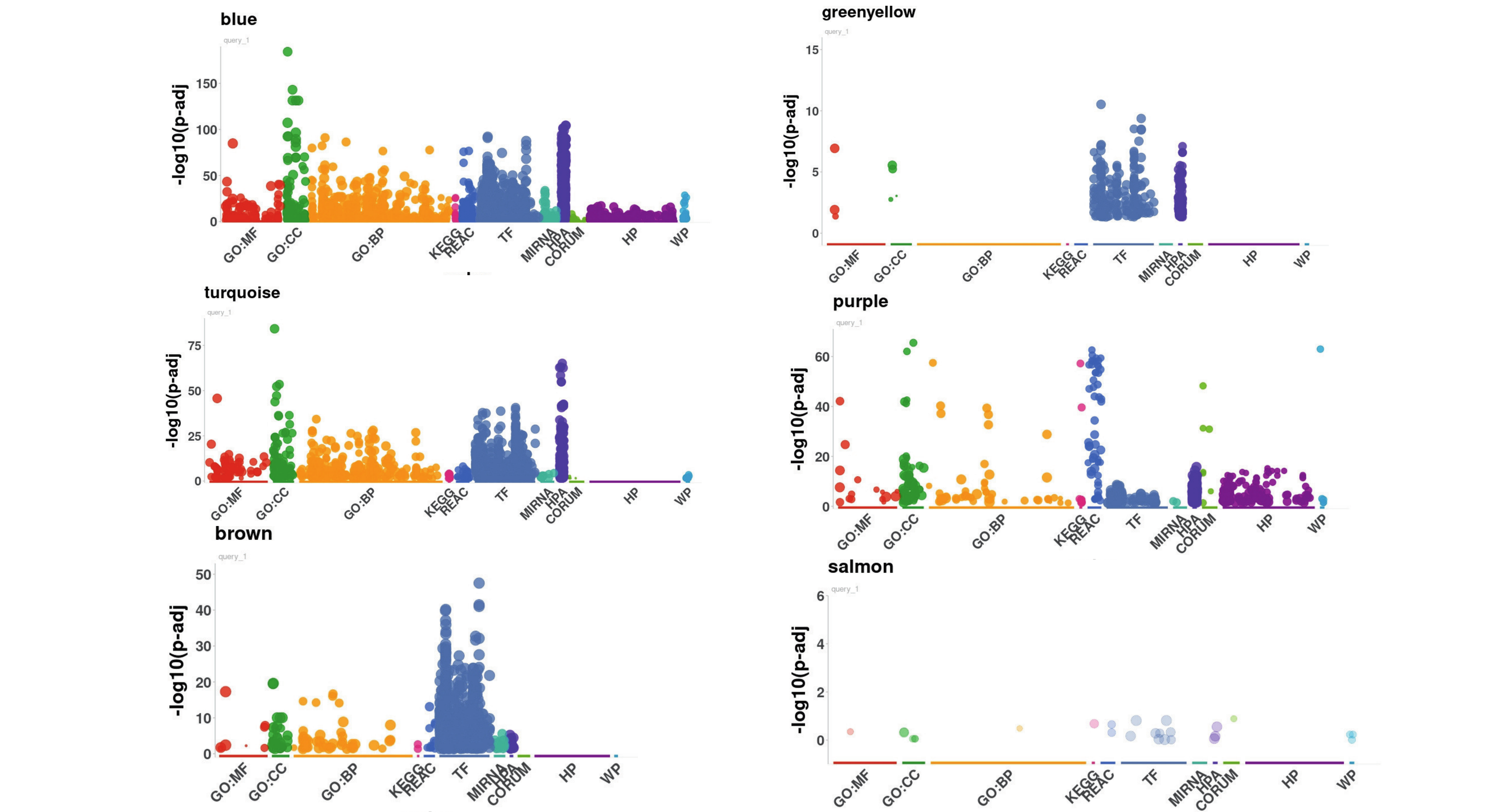

Supplement: Supplementary file 1 — Figure S1. [file CAM4-13-e6945-s003.png]

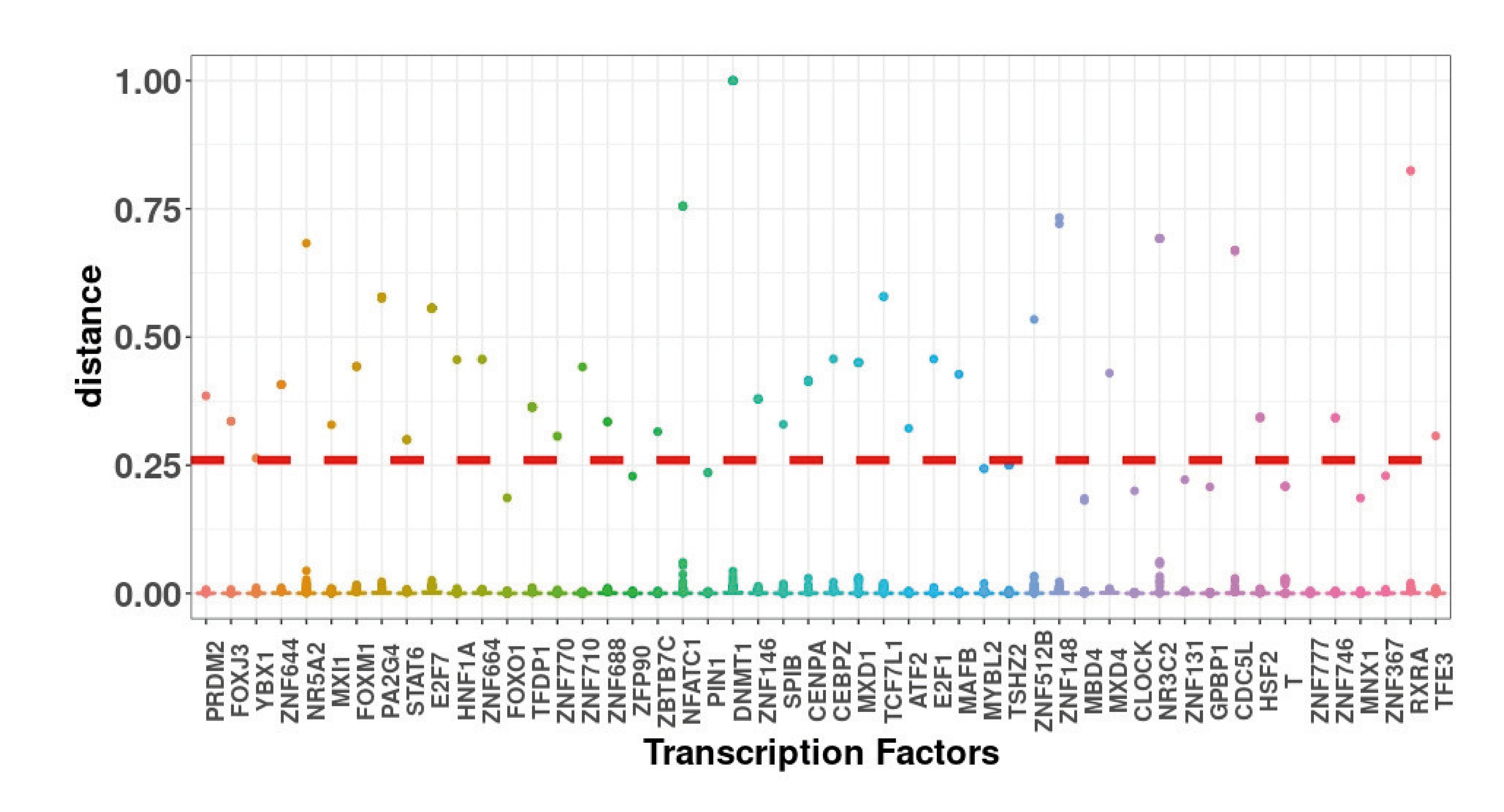

Supplement: Supplementary file 2 — Figure S2. [file CAM4-13-e6945-s002.png]

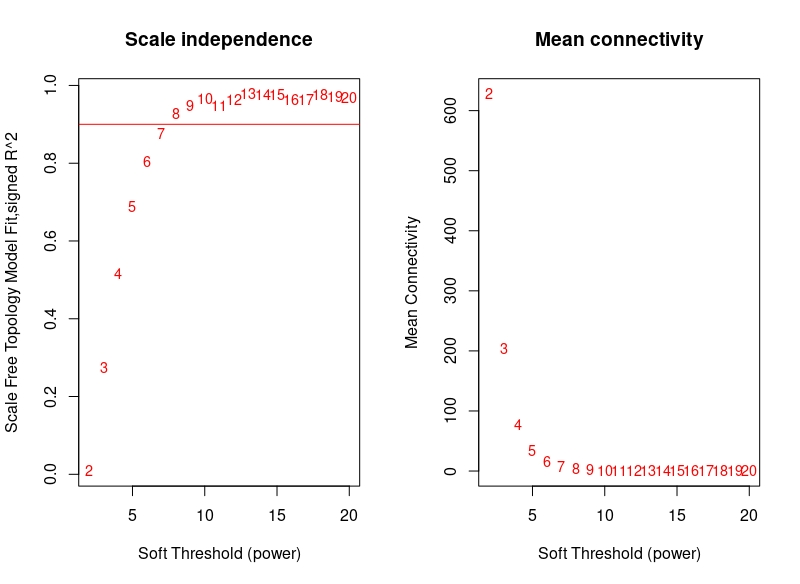

Supplement: Supplementary file 3 — Figure S3. [file CAM4-13-e6945-s001.png]
